# Molecular characterization of Serrasalmidae hybrid in the upper Paraná River floodplain using molecular markers

**DOI:** 10.1111/jfb.70101

**Published:** 2025-05-27

**Authors:** Laura Ivana Ramos, Eloisa de Páris Paz, Lidiany Doreto Cavalcanti, Ricardo Massato Takemoto, Alessandra Valéria de Oliveira

**Affiliations:** ^1^ Programa de Pós‐graduação em Ecologia de Ambientes Aquáticos Continentais, Centro de Ciências Biológicas Universidade Estadual de Maringá Maringá Brazil; ^2^ Núcleo de Pesquisas em Limnologia, Ictiologia e Aquicultura (Nupélia), Centro de Ciências Biológicas Universidade Estadual de Maringá Maringá Brazil; ^3^ Departamento de Biotecnologia, Genética e Biologia Celular, Centro de Ciências Biológicas Universidade Estadual de Maringá Maringá Brazil

**Keywords:** genetic introgression, genetic monitoring, species introduction

## Abstract

This study reports the molecular identification of a tambacu—a hybrid of *Colossoma macropomum* and *Piaractus mesopotamicus*—in the floodplain of the upper Paraná River using molecular markers. The PCR‐GEL method, based on the analysis of *COI* and *TROP* fragments, successfully characterized the hybrid, while PCR‐SEQ, employing *RAG2* and *TROP* markers, identified variable sites that differentiate the parental species and the hybrid.

Family Serrasalmidae, native to South American rivers, is commercially valuable and ranks as the second most‐produced fish family in Brazil (PeixeBR, 2024). Fish farms frequently produce hybrids within this family (IBAMA, 2007). *Colossoma macropomum* (Cuvier, 1816), commonly known as tambaqui, is often crossbred with other species, such as *Piaractus brachypomus* (Cuvier, 1818) (pirapitinga) and *Piaractus mesopotamicus* (Holmberg, 1887) (pacu). Hybrid production raises significant environmental concerns because escapes or intentional releases during stocking are major contributors to their introduction into natural habitats (EMBRAPA, 2014). Morphological analyses do not always provide reliable identification of hybrids (Mallet, 2005). Molecular methods provide accurate and highly specific identification of fish species and their hybrids, complementing morphological data (Hashimoto et al., 2011). This report documents the molecular characterization of a tambacu—a hybrid of *C. macropomum* (female) and *P. mesopotamicus* (male)—in the floodplain of the upper Paraná River using molecular markers.

The specimen analysed was obtained from fishermen and initially identified as *P. mesopotamicus*. It was collected in May 2023 on Ilha Mineira (22°41′39.4″S, 53°09′25.8″W) in the floodplain of the upper Paraná River (Data S1). DNA was extracted from muscle tissue using the Wizard® Genomic DNA Purification Kit (Promega), following the manufacturer's protocol. Regions of the cytochrome *c* oxidase subunit I (*COI*), α‐tropomyosin (*TROP*) and recombination activation gene 2 (*RAG2*) were amplified from the sample (Data S2). Two methods of analysis were employed: gel analysis (PCR‐GEL) and sequencing (PCR‐SEQ). For PCR‐GEL, after amplification, the fragments generated were visualized on a 2.5% agarose gel to determine their size, with a 100 bp ladder marker as a reference. Fragment sizes were compared with data reported by Hashimoto et al. (2011), where fragments of 435 bp (for *COI*) and 172 bp (for *TROP*) correspond to *C. macropomum* and fragments of 307 bp (*COI*) and 269 bp (*TROP*) correspond to *P. mesopotamicus*. For PCR‐SEQ, the PCR products were purified and sequenced. *C. macropomum* and *P. mesopotamicus* sequences generated by Hashimoto et al. (2011) were retrieved from GenBank for comparison with the sequences of this study. The sequences were edited and aligned by Clustal W (Thompson et al., 1994) in BioEdit 7 (Hall, 1999) and MEGA 7 (Kumar et al., 2016), respectively. Visual analysis of the sequences was conducted to identify polymorphic sites and allelic differences. The sequences obtained were deposited in GenBank under accession numbers PQ375112 (*RAG2*) and PQ375113 (*TROP*).

Analysis of the *COI* mitochondrial marker (Figure 1a) revealed that specimen H_1_ exhibited a fragment of approximately 435 bp, a fragment size corresponding to that of *C. macropomum* as reported by Hashimoto et al. (2011), suggesting a maternal origin of the hybrid. For the *TROP* nuclear marker (Figure 1b), DNA fragments of approximately 269 and 172 bp were amplified, the fragment sizes corresponding to those of *P. mesopotamicus* and *C. macropomum* as reported by Hashimoto et al. (2011), indicating that specimen H_1_ is an F_1_ hybrid.

**FIGURE 1:**
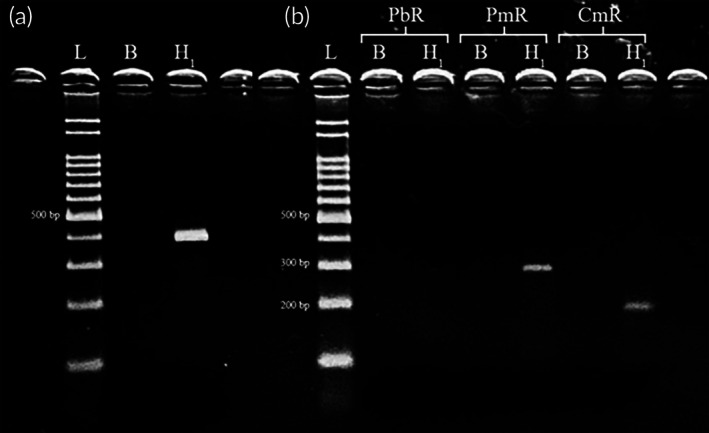
Electrophoretic analysis of the DNA fragments obtained for the specimen analysed (H_1_) with the species‐specific primers for the *COI* region (a) and for the nuclear gene *TROP* (b). The primer TROP PbR specific for *Piaractus brachypomus* (pirapitinga), TROP PmR specific for *P. mesopotamicus* (pacu) and TROP CmR specific for *Colossoma macropomum* (tambaqui). L, 100 bp molecular weight marker; B, negative control.

Sequences of *RAG2* (684 bp) and *TROP* (256 bp) were visually analysed, and a table was created to compare polymorphic sites among *P. mesopotamicus*, *C. macropomum* and the H_1_ hybrid specimen (Table 1). For the *RAG2* marker, 19 polymorphic sites were identified that distinguish the two species, with the H_1_ hybrid displaying polymorphisms unique to both *P. mesopotamicus* and *C. macropomum* at all sites. Regarding the *TROP* marker, among the 13 variable regions that differentiate the species, only two (sites 51 and 228) were detected simultaneously in the H_1_ hybrid. The 207 site exhibited an overlapping peak R (overlay of G and A). In the remaining 10 regions, the H_1_ hybrid exhibited the molecular profile of *C. macropomum*.

**TABLE 1:** Variable sites of the nuclear molecular markers *RAG2* and *TROP* for Serrasalmidae species and the GenBank access code. Variable sites are determined based on the alignment of the samples.

Sample	Specie	Variable sites																																GenBank access	
		*RAG2*																			*TROP*													*RAG2*	*TROP*
		12	124	146	157	176	185	270	304	377	404	431	437	440	461	521	654	656	567	679	51	75	76	77	78	79	80	81	82	117	119	207	228		
CEPTA001	*P. mesopotamicus*	G	A	A	A	G	G	T	G	A	C	C	C	G	T	G	G	T	G	G	G	G	C	A	T	A	A	A	G	G	T	T	A	HQ420863	HQ420878
CEPTA002	*P. mesopotamicus*	G	A	A	A	G	G	T	G	A	C	C	C	G	T	G	G	T	G	G	G	G	C	A	T	A	A	A	G	G	T	T	A	HQ420864	HQ420879
CEPTA003	*P. mesopotamicus*	G	A	A	A	G	G	T	G	A	C	C	C	G	T	G	G	T	G	G	G	G	C	A	T	A	A	A	G	G	T	T	A	HQ420865	HQ420880
CEPTA004	*P. mesopotamicus*	G	A	A	A	G	G	T	G	A	C	C	C	G	T	G	G	T	G	G	G	G	C	A	T	A	A	A	G	G	T	T	A	HQ420866	HQ420881
CEPTA004	*P. mesopotamicus*	G	A	A	A	G	G	T	G	A	C	C	C	G	T	G	G	T	G	G	G	G	C	A	T	A	A	A	G	G	T	T	A	HQ420867	HQ420882
CEPTA011	*C. macropomum*	A	G	G	T	A	A	C	C	G	T	T	T	A	C	T	A	C	A	A	A	‐	‐	‐	‐	‐	‐	‐	‐	A	C	G	G	HQ420873	HQ420888
CEPTA090	*C. macropomum*	A	G	G	T	A	A	C	C	G	T	T	T	A	C	T	A	C	A	A	A	‐	‐	‐	‐	‐	‐	‐	‐	A	C	G	G	HQ420874	HQ420889
CEPTA091	*C. macropomum*	A	G	G	T	A	A	C	C	G	T	T	T	A	C	T	A	C	A	A	A	‐	‐	‐	‐	‐	‐	‐	‐	A	C	G	G	HQ420875	HQ420890
CEPTA092	*C. macropomum*	A	G	G	T	A	A	C	C	G	T	T	T	A	C	T	A	C	A	A	A	‐	‐	‐	‐	‐	‐	‐	‐	A	C	G	G	HQ420876	HQ420891
CEPTA093	*C. macropomum*	A	G	G	T	A	A	C	C	G	T	T	T	A	C	T	A	C	A	A	A	‐	‐	‐	‐	‐	‐	‐	‐	A	C	G	G	HQ420877	HQ420892
H_1_	F_1_ hybrid	R	R	R	W	R	R	Y	S	R	Y	Y	Y	R	Y	K	R	Y	R	R	R	‐	‐	‐	‐	‐	‐	‐	‐	A	C	R	R	PQ375112	PQ375113

Abbreviations: R, overlapping bases G and A; W, overlapping bases A and T; S, overlapping bases A and G; Y, overlapping bases T and C; K, overlapping bases G and T.

The production of Serrasalmidae hybrids is frequently conducted on aquaculture farms and is considered a technological advancement in the sector. This practice is justified by its reduction of commercial fishing impact on natural populations and its production of animals with characteristics better suited to market demands (Hashimoto et al., 2011). However, a significant drawback is the introduction of these hybrids into natural environments, primarily through escapes from rearing tanks due to breakage, overflowing, emptying or management activities (Hashimoto et al., 2011). Regarding hybrids, two scenarios emerge. First, first‐generation hybrids (F_1_) are typically sterile or unviable. While these pose no genetic risk, they present ecological risks as effective competitors that may destroy developmental habitats and reduce reproductive success (Fitzpatrick et al., 2015). The second scenario involves fertile hybrids, as observed in Serrasalmidae. These hybrids, beyond being competitors and causing ecological impacts, can cause genetic introgression. Such events affect wild populations and, in extreme cases, may lead to species extinction (Fitzpatrick et al., 2015). Subsequent generations after introgression may exhibit severe deformities due to improper pairing of non‐homologous chromosomes (Hashimoto et al., 2012), as well as reduced fertility, growth rates and survival due to inbreeding depression (Charlesworth & Willis, 2009).

Previous molecular marker studies identified a hybrid individual of *P. brachypomus* and *C. macropomum*, originating from aquaculture escapes, in the Teles Pires River of the Amazon Basin (Lima et al., 2019). Other fish hybridization events have also been reported in the Paraná River basin (Casimiro et al., 2018; Cavalcanti et al., 2024). The tambacu hybrid studied here could not exist naturally without human intervention, as its parent species have distinct natural distributions: *P. mesopotamicus* is native to the Paraná‐Paraguay basin, while *C. macropomum* is native to the Amazon basin (Orsi & Agostinho, 1999) (Data S1). While determining the exact cause of these hybrids' presence in the wild is challenging, aquaculture is considered the primary driver of exotic species introductions in Neotropical reservoirs (Ortega et al., 2015). Casimiro et al. (2018) estimated that approximately 1.14 million fish at various growth stages escaped during floods in the 2015 and 2016 rainy seasons in the Lower and Middle Paranapanema River Basin. These escapes included seven native species, 14 non‐native species and three hybrid varieties, including tambacu. The absence of studies analysing eggs and larvae in this habitat makes it difficult to determine whether interspecific crosses are occurring in the wild. However, data from Cavalcanti et al. (2024) indicate a higher prevalence of hybrids between *P. mesopotamicus* and *P. brachypomus* compared to pure species in the upper Paraná River basin, suggesting such events may be occurring.

Molecular diagnostic tools are valuable for monitoring hybridization and could enhance aquaculture production while preserving the genetic integrity of both farmed and native populations.The PCR‐GEL method proved effective for characterizing the hybrid status of specimen H1. Among the PCR‐SEQ markers, RAG2 was particularly informative, revealing 19 polymorphic sites that clearly distinguished P. mesopotamicus and C. macropomum, with H_1_ exhibiting diagnostic positions from both parental species across all sites—strongly supporting its hybrid origin. Given the commercial importance of hybrids in fish farming and the ecological risks posed by their introduction into natural environments, implementing specific and stringent legislation to regulate hybrid production and prevent accidental escapes is crucial (Nobile et al., 2020).

Permission to access the genetic heritage of these species was obtained through the Sistema Nacional de Gestão do Patrimônio Genético (SISGEN; registration A1C1E26). This research was funded by the Conselho Nacional de Desenvolvimento Científico e Tecnológico (CNPq), with infrastructure support provided by the Núcleo de Pesquisa em Limnologia, Ictiologia e Aquicultura (Nupélia) and the Nupélia Molecular Genetics Laboratory (Nupgen). The authors would like to thank Master Bruno Henrique Mioto Stabile for his assistance in preparing the geographic map.

## AUTHOR CONTRIBUTIONS

All authors contributed to the conception and design of the study. Molecular analyses were performed by Laura I. Ramos, Eloisa de P. Paz, and Alessandra V. de Oliveira. All authors contributed to the writing of the manuscript and read and approved the final version.

## Supporting information


**DATA S1** Map of the collection region of the H_1_ hybrid, represented by the red circle, and the native distribution of its parental species: the Amazon basin (orange) for *C. macropomum* and the Paraná‐Paraguay basin (green) for *P. mesopotamicus*.

**DATA S2** Molecular methods used for hybridization analysis in Serrassalmidae, with specific primers and amplification conditions.
