# Supplementary figures and images for: Molecular characterization of Serrasalmidae hybrid in the upper Paraná River floodplain using molecular markers
Source: J Fish Biol. 2025 May 27;107(3):1067–70. doi: 10.1111/jfb.70101 (PMC12463763; doi:10.1111/jfb.70101)

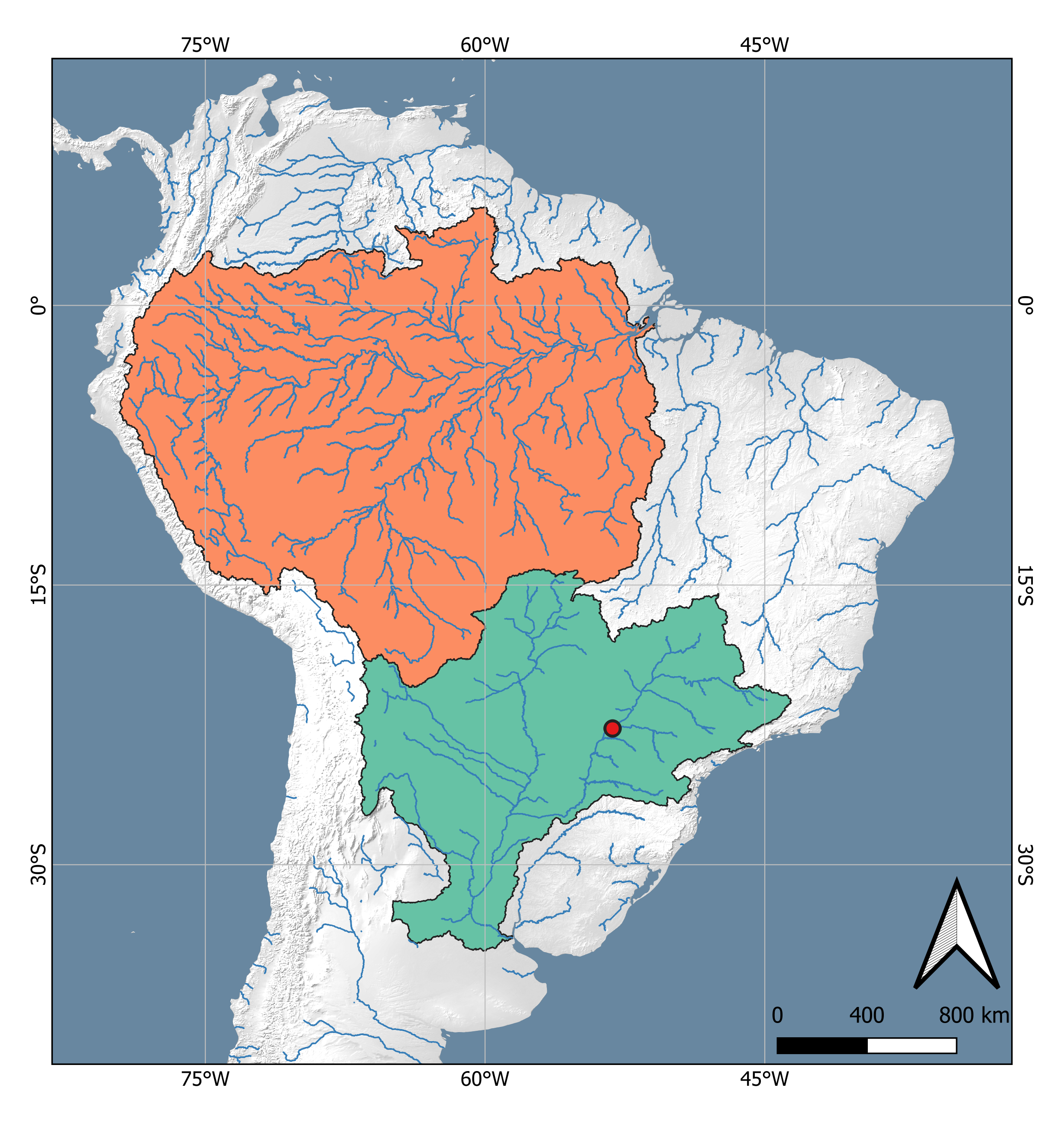

Supplement: Supplementary file 1 — DATA S1 Map of the collection region of the H1 hybrid, represented by the red circle, and the native distribution of its parental species: the Amazon basin (orange) for C. macropomum and the Paraná‐Paraguay basin (green) for P. mesopotamicus. [file JFB-107-1067-s002.tif]
