# Supplementary material for: Molecular characterization of Serrasalmidae hybrid in the upper Paraná River floodplain using molecular markers
Source: J Fish Biol. 2025 May 27;107(3):1067–70. doi: 10.1111/jfb.70101 (PMC12463763; doi:10.1111/jfb.70101)
Supplement: Supplementary file 2 — DATA S2 Molecular methods used for hybridization analysis in Serrassalmidae, with specific primers and amplification conditions. [file JFB-107-1067-s001.docx]

**Supplementary Material 2.** Molecular methods used for hybridization analysis in Serrassalmidae, with specific primers and amplification conditions.

| **Method** | **Gene** | **Primer sequence (5' 3')** | **PCR conditions (35 cycles)** |
| --- | --- | --- | --- |
| PCR-GEL | *COI* | [COI F (TCAACCAACCACAAAGACATTGGCAC)](https://www-sciencedirect.ez79.periodicos.capes.gov.br/science/article/pii/S0044848611006429#tf0005) | 30s at 95 °C, 30s at 50 °C and 45s at 72 °C |
|  |  | COI CmR (AGCAAGATGGAGTGAGAAAATA) |  |
|  |  | COI PbR (CTGTCAGAAGTATAGTAATTCCG) |  |
|  |  | COI PiaractusR (GAAGGAAGGATGGGGGTAGG) |  |
|  | *TROP* | [TROP F (GAGTTGGATCGGGCTCAGGAGCG)](https://www-sciencedirect.ez79.periodicos.capes.gov.br/science/article/pii/S0044848611006429#tf0010) | 30s at 95 °C, 30s at 60 °C and 10s at 72 °C |
|  |  | TROP CmR (ATACAACAATGCCATCGCT) |  |
|  |  | TROP PmR (CTTCAGCTGGATCTCCTGA) |  |
|  |  | TROP PbR (TTGACTTTATGCCACACAAAT) |  |
| PCR-SEQ | *RAG2* | RAG2 R (GTGGCTCTCGAGGTTCCATA) | 30s at 95 °C, 30s at 55 °C and 45s at 72 °C |
|  |  | RAG2 F (AGCTGCGTGCCATTCATTCT) |  |
|  | *TROP* | [TROP R (CGGTCAGCCTCTTCAGCAATGTGCTT)](https://www-sciencedirect.ez79.periodicos.capes.gov.br/science/article/pii/S0044848611006429#tf0010) | 30s at 95 °C, 30s at 60 °C and 10s at 72 °C |
|  |  | TROP F (GAGTTGGATCGGGCTCAGGAGCG) |  |
